# Role of Natural Cross Linkers in Resin–Dentin Bond Durability: A Systematic Review and Meta-Analysis

**DOI:** 10.3390/ma15165650

**Published:** 2022-08-17

**Authors:** Lavanya Anumula, Sindhu Ramesh, Venkata Suneel Kumar Kolaparthi, Richard Kirubakaran, Mohmed Isaqali Karobari, Suraj Arora, Ahmed A. Saleh, Omir Aldowah, Pietro Messina, Giuseppe Alessandro Scardina

**Affiliations:** 1 Department of Conservative Dentistry and Endodontics, Narayana Dental College and Hospital, Nellore 524003, Andra Pradesh, India; 2 Department of Conservative Dentistry and Endodontics, Saveetha Dental College and Hospital, Chennai 600077, Tamil Nadu, India; 3 Department of Oral Medicine and Radiology, Narayana Dental College and Hospital, Nellore 524003, Andra Pradesh, India; 4 Cochrane South Asia, BV Moses Centre for Evidence Informed Health Care and Health Policy, Christian Medical College, Vellore 632004, Tamil Nadu, India; 5 Conservative Dentistry Unit, School of Dental Sciences, Universiti Sains Malaysia, Health Campus, Kubang Kerian, Kota Bharu 16150, Malaysia; 6 Department of Restorative Dentistry & Endodontics, Faculty of Dentistry, University of Puthisastra, Phnom Penh 12211, Cambodia; 7 Department of Restorative Dental Sciences, King Khalid University, P.O. Box 960, Abha 61421, Saudi Arabia; 8 Prosthetic Dental Science Department, Faculty of Dentistry, Najran University, Najran 11001, Saudi Arabia; 9 Department of Surgical, Oncological and Stomatological Disciplines, University of Palermo, 90133 Palermo, Italy

**Keywords:** matrix metalloproteinase, matrix metalloproteinase inhibitors, plant extract, Grape seed extract, flavonoids

## Abstract

Background: The role of endogenous Matrix Metallo Proteinases in resin dentin bond deterioration over time has been well documented. The present study aimed to systematically review the literature; in vitro and ex vivo studies that assessed the outcomes of natural cross-linkers for immediate and long-term tensile bond strength were included. Methods: The manuscript search was carried out in six electronic databases—PubMed/MEDLINE, LILACS, SciELO, Cochrane, Web of Science and DOAJ, without publication year limits. Only manuscripts in English (including the translated articles) were selected, and the last search was performed in December 2020. The Preferred Reporting Items for Systematic Reviews and Meta-Analyses (PRISMA) statement was followed. Results: From the 128 potentially eligible studies, 48 full-text articles were assessed for eligibility. After eligibility assessment and exclusions, 14 studies were considered for systematic review and seven studies for meta-analysis. Amongst the selected studies for meta-analysis, three had a medium and four had a low risk of bias. Conclusions: It was evidenced by the available data that Proanthocyanidin is the most efficient natural cross-linker to date, in preserving the bond strength even after ageing.

## 1. Introduction

The composite restorative material, along with contemporary adhesives and techniques, has revolutionized aesthetic dentistry practices. The success of a composite restoration entirely depends upon the durability of dentin bonding. The collagen matrix plays a vital role in maintaining the integrity and stability of the hybrid layer thus improving the mechanical properties and durability of bonding [1,2].

Many studies have demonstrated mechanical and ultrastructural disruptions of the hybrid layer after a certain period [3,4,5,6] and thus identified the limited durability of the hybrid layer among in vivo and in vitro studies. This consequence could be due to a myriad of factors such as deficient resin monomer infiltration of demineralized dentin and elution of unpolymerized monomers from polymerized adhesives. These exposed collagen fibres (incompletely infiltrated) within the hybrid layer may become susceptible to degradation [7]. The endogenous Matrix Metallo Proteinases (MMP) are present in the dentin in the dormant form [8] and are triggered in a low pH environment comparable to the etching and carious process [9]. These host-derived proteases slowly degrade collagen fibres and play a significant role in the destruction of the bonded surface accounting for up to 36–70% bond strength loss after 12 to 14 months [10]. The bond strength gradually decreases and it has been proven in vitro, when the samples were tested after 3 months [11] or 100 days of storage [4]. The weakening of the bond may be attributed to the displacement of adhesive by water at the resin–dentin contact as a result of hydrolysis [4,11]. There is adequate evidence to disprove the role of bacteria in collagenolytic/gelatinolytic activity in dentin demineralized with etch-and-rinse adhesives [12,13], thus supporting the probable association of host-derived proteases in the degradation of incompletely-infiltrated collagen fibrils within hybrid layers [3,5,6,13]. Thus it has been the holy grail of researchers to achieve a durable bond with a sustainable hybrid layer that would last a considerable time.

The new endeavour in adhesive dentistry has been to inhibit the MMPs or stabilize collagen, as etching is an inevitable step. Both natural and synthetic materials have been tested for improving bond strength and durability. Due to biosafety, clinical feasibility and effectiveness, the naturally derived materials have attracted significant attention [14]. Many natural products such as Proanthocyanidin (Grape seed extract and cocoa seed extract) [1,15,16,17,18,19], Green tea [20,21,22], Epigallocatechin gallate (EGCG) [20,23], Biocalein [14,24,25], Quercetin [26,27,28,29], Naringin [26,28], Cardol andCardinal [30], Aroeira [30] etc. have been tested as collagen crosslinkers and MMP inhibitors. As most natural products have proven to stabilise the collagen along with MMP inhibition, they are referred to as “collagen cross-linkers” in this study and thus the title. The cross-linking agents when applied on demineralised dentin, inactivate the catalytic site of the proteolytic enzymes thus preventing the disruption of the hybrid layer. These agents have either been used after the acid etching process (pre-treatment liner) or incorporated into the adhesive system. The improvisation of collagen cross-links with these natural agents has been proposed to improve the mechanical stability and reduce the biodegradation rates of collagen, thereby increasing the dentin resin bond durability. Hence this can be an effective way to increase the clinical longevity of the restoration. The use of these alternative natural cross-linkers has increased in the last decade and there is enormous data in the literature regarding the same. A systematic review and meta-analysis by Montagner et al. [31] and O.Kiuru et al. [32] indicated that chlorhexidine (CHX) is effective in inhibiting the collagen degrading enzyme thus maintaining long term bond strength. However, there has hitherto been no review reporting the influence of the natural cross-linkers in the literature.

Thus, this study aimed to systematically review the literature for in vitro and ex vivo studies to evaluate the effect of natural cross-linkers on resin–dentin bond strength immediately after bonding and after ageing, thus testing the durability of the bond. On examination of the manuscripts regarding the effect of cross-linkers on micro tensile bond strength, there were contradictory results; i.e., a few showed an improvement in bond strength and a few studies did not show any improvement. Thus the null hypothesis tested in this study was that the use of natural cross-linkers does not affect the bond strength values and its durability.

## 2. Material and Methods

The PRISMA statement for reporting systematic reviews and meta-analyses was followed in conducting this systematic review and meta-analysis [33].

The following PICOS criteria were followed to frame the search terms:

P (Problem/Population): The eventual resin–dentin bond degradation.

I (Intervention): Use of natural MMP inhibitors/Crosslinkers.

C (Comparison): Without the use of inhibitors.

O (Outcome): Durability of the resin–dentin bond measured by the micro tensile bond test. (To evaluate if the use of crosslinker in demineralized dentin increases the stability of resin–dentin bond immediately and in a long term period).

S (Study design): In vitro or ex vivo studies.

### 2.1. Information Sources and Eligibility Criteria

The manuscript search was carried out in six electronic databases—PubMed/MEDLINE, LILACS, SciELO, Cochrane, Web of Science and DOAJ. Only manuscripts in English (including the translated articles) were selected, and the last search was performed in Dec 2020. The PICO strategy was used as the basis for this research, using the appropriate descriptors. The search words were as follows: (collagen degradation) OR MMP) OR dentin proteases) OR hydrolysis of the hybrid layer) AND (natural extract*) OR herbal products) OR plant extract) OR mmp inhibitors) OR natural cross-linking agent) OR collagen cross-linking) AND (dentin*adhesive) OR adhesive system*) AND (stability of hybrid layer) OR stability of resin dentin bond) OR resin dentin bond strength) OR micro tensile bond strength).

### 2.2. Types of the Study Included

In vitro studies or ex vivo studies that evaluated the efficacy of natural cross linkers/MMP inhibitors (MMPI) during the adhesive step were included.

To be included, the studies had to fulfil the following criteria: in vitro studies, outcome measured in terms of micro tensile bond strength (Megapascal), plant derivatives/extracts used as a crosslinker/MMPI, comparative studies with at least one control group without the use of cross linkers/MMPI and ageing performed either in artificial saliva or distilled water.

The predetermined rejection criteria were the inclusion of bovine teeth, collagenase ageing and application of Crosslinker/MMPI before the etching step.

### 2.3. Data Collection Process: Screening and Selection—Was Performed in 4 Steps

Step 1: Two authors (L.A and S.R) independently reviewed the titles and abstracts based on the search strategy. Step 2: The abstracts were reviewed by two authors (L.A and S.R) independently and were selected based on the inclusion criteria. If consensus was not reached, the abstract was set aside for further evaluation. Step 3: Full-text articles of the abstracts selected in step 2 were retrieved and reviewed by both authors. Any disagreements among the authors were discussed and resolved by the third author (V.S.K). Step 4: The references of all the included articles in Step 3 were manually searched for further relevant studies that could fulfil the inclusion criteria, and potentially interesting articles were examined.

### 2.4. Data Extraction

A protocol for data extraction was defined and evaluated by both the authors (L.A and S.R). The included studies were divided based on the natural cross linker used. The data was extracted from the full-text articles by one author (L.A) and re-examined by the second author (S.R).

A customized electronic spreadsheet was specifically designed to record the data extracted from the articles mentioned above. The data sheet included the information of the authors, year of publication, the samples tested (n), the natural MMP inhibitor, its concentration, the period of application of the test material, the type of adhesive system used (total etch or self etch), ageing period, the solution used for ageing, the testing period (6 months or 12 months or more), the other materials tested and the data of the micro tensile bond strength expressed in megapascals. Some studies did not report the precise bond strength values, and results were represented in graphs or figures. The authors of such studies were contacted through mail, requested data, and obtained information [21,23]. The studies were excluded from the review when the authors did not provide the data even after emailing them twice.

### 2.5. Qualitative Assessment

The risk of bias was evaluated independently by the two reviewers, considering the aspects reported in the materials and methods sections of the articles. The assessment was based on the previous review [34], and the following parameters were considered for quality assessment: specimen randomisation; teeth free of caries; specimens with similar dimensions; materials used according to manufacturer’s instructions; sample size calculation; blinding of the operator of the testing machine; sample preparation and handling; application of test material and specimen test according to standard specifications. If the author reported the parameter in the article, it was given a “Y” (yes) on that specific parameter, and if the information was not found in the article, then it was allotted an “N” (no). Every Y would count as 1 (one) entity. Articles that reported one to four items in the articles were categorized as high risk of bias, five to six items as medium risk of bias and seven to nine items as low risk of bias.

### 2.6. Data Analysis

**Figure 1:**
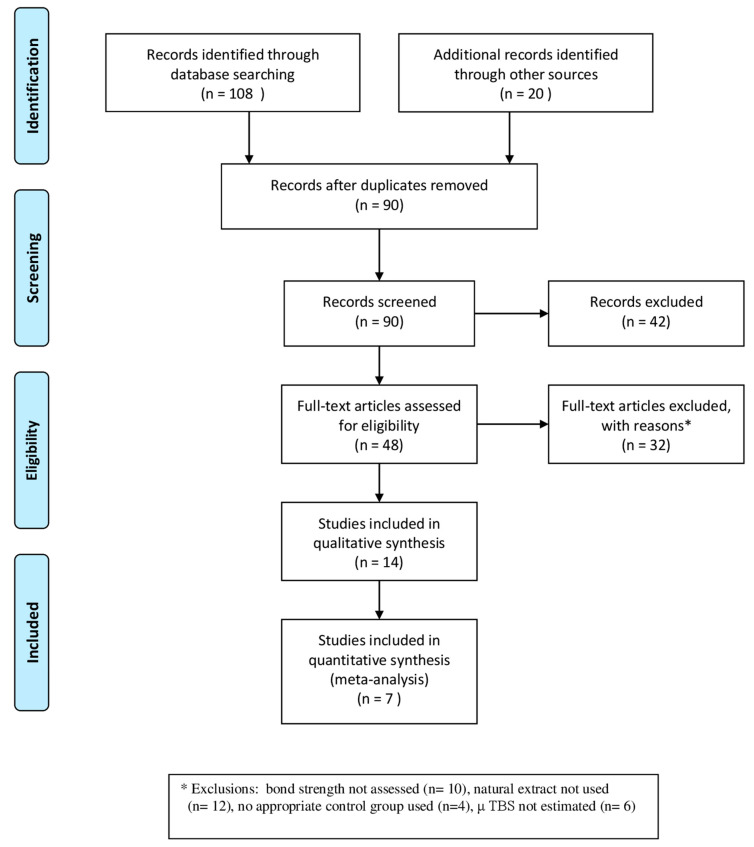
Flow diagram of study selection according to PRISMA statement.

**Figure 2:**
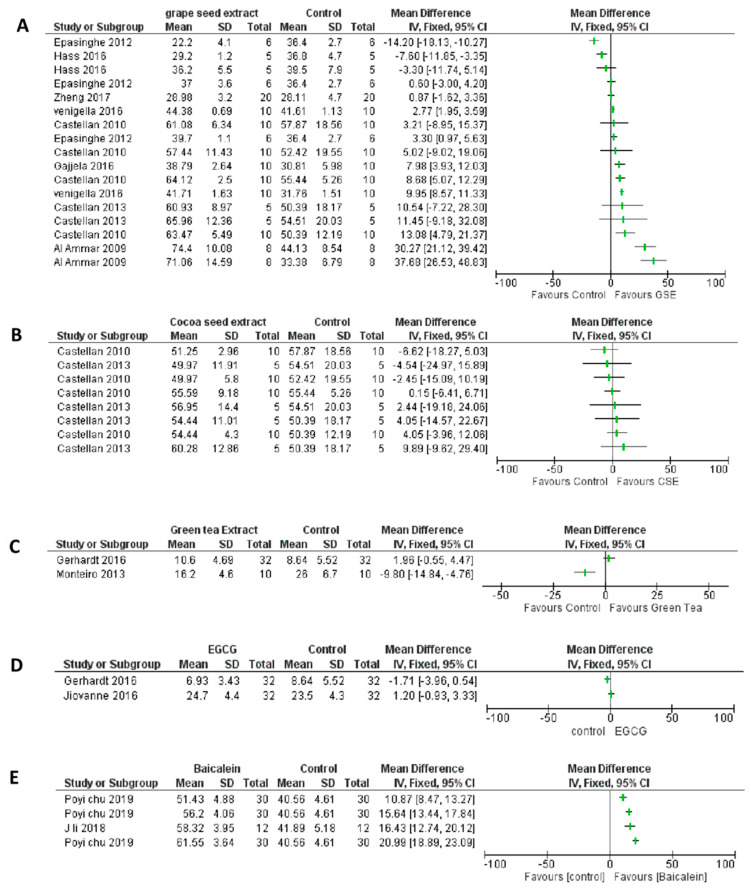
Forest plot analysis of studies at baseline (No ageing). (**A**)—Proanthocyanidin (PA)/Grape seed extract (GSE) vs. control at baseline. (**B**)—Cocoa seed extract (CSE) vs. control at baseline. (**C**)—Green tea extract (GTE) vs. control at baseline. (**D**)—Epigallocatechin gallate (EGCG) vs. control at baseline. (**E**)—Baicalein vs. control at baseline.

Out of the total 14 articles selected for systematic review (Figure 1), five different natural cross linkers were evaluated (Table 1). It was judged, keeping the baseline data at observation, that the difference in the natural MMP inhibitors with various concentrations, different adhesives (total-etch and self-etch) and soaking time would not contribute to meaningful conclusions from a statistically pooled result. Therefore, a meta-analysis was not conducted for the data obtained at baseline and is presented in a forest plot (Figure 2A–E), and patterns/trends of the data were studied.

**Figure 3:**
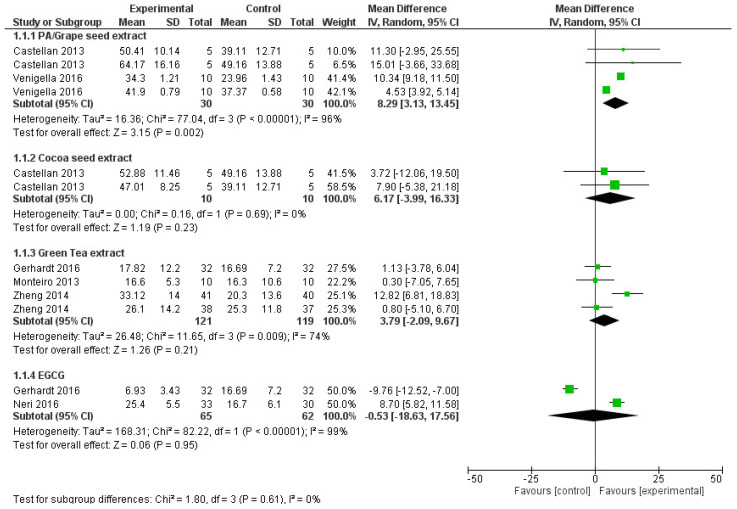
Forest plot analysis of studies with 6–9 months of ageing (<1 year).

**Figure 4:**
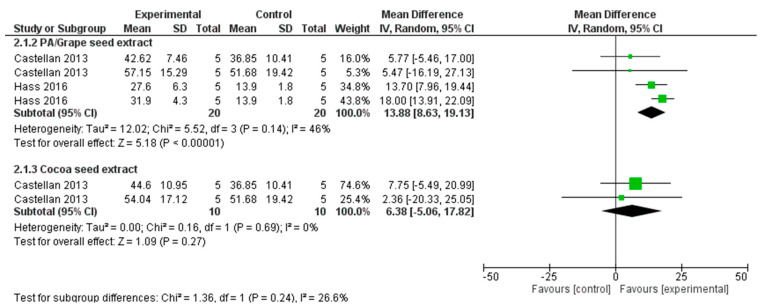
Forest plot analysis of studies with 12 months of ageing (>1 year).

For the meta-analysis (Figure 3 and Figure 4), only the data from the studies that used natural cross-linker and were aged more than or at least six months were included. Statistical heterogeneity of the treatment effect among studies was assessed via the Cochran Q test, with a threshold *p*-value of 0.1, and the inconsistency I^2^ test values > 50% were considered indicative of high heterogeneity [40]. The number of specimens was viewed as the number of experimental units. All the results/analyses from each study were conducted with Review Manager Software 5.3 (Review Manager (RevMan) [Computer program]. Version 5.3. Copenhagen: The Nordic Cochrane Centre, The Cochrane Collaboration, 2014). A random effects model with a mean difference to measure the effect size among studies was adopted.

## 3. Results

In total, 128 potentially eligible studies were obtained from various databases and other resources. Out of which, 48 full-text articles were assessed for eligibility. After eligibility assessment and exclusions, 14 studies were selected for systematic review and seven studies were included for meta-analysis, all of which were in vitro studies (Figure 1) [1,19,20,21,23,35,38]. The most commonly used natural MMP inhibitor was proanthocyanidin (6.5%), and the most used ageing protocol was artificial saliva and distilled water. The characteristics of the included studies are provided in Table 2.

### Risk of Bias

Of the seven studies included for meta-analysis, four presented a low risk of bias, and three studies showed medium risk. Table 3 represents the risk of bias and the factors considered for the analysis.

From the included studies, only the data of interest were extracted. For instance, in the study in which the natural MMP inhibitor was mixed in the phosphoric acid [18], the micro tensile bond strength was tested without the application of adhesive and composite restoration [41], without the inclusion of control group [26,27,42], and ageing of the samples performed with collagenase [29], the data were not extracted. The study by Fang et al. [43] was not included in the review as it had too many variables, the tested material was applied for 1 h, and the micro tensile bond strength (µTBS) was tested immediately after bonding. The immediate testing of the samples is usually performed after the storage of the samples in artificial saliva/distilled water for 24 h. Quantitative analysis: tested at baseline (Tested after 24 h): Figure 2A–E.

The analysis was performed with 33 datasets although only 14 studies were included. All the results are described narratively in the following order.

1. Proanthocyanidin (PA)/Grape seed extract (GSE) vs. control at baseline;
2. Cocoa seed extract (CSE) vs. control at baseline;
3. Green tea extract (GTE) vs. control at baseline;
4. Epigallocatechin Gallate (EGCG) vs. control at baseline;
5. Baicalein vs. control at baseline.

PA/GSE vs. control: eight studies evaluated PA (Table 1). The majority (12/17 (70%)) of the reports showed a positive trend and the effect estimate was in favour of PA. There were many variations in the studies, including the concentration of the cross linker, PA in the adhesive [16], ethanol/water-wet bonding, and different soaking/treatment times. There was an increased variation in the result when PA was added to the adhesive.

CSE vs. control: 50% of the reports (4/8) show a positive trend, though not very evident. The soaking time of the samples in the test material, bonding agent and preparation of the extract (solvent used) varied among the studies.

GTE vs. control: In the two studies observed for immediate bond strength, a wide variation was observed, and only one study favoured the use of GTE.

EGCG: When the patterns of the two studies were observed, it was noticed that none of them showed a favourable result.

Baicalein: All four reports (100%) showed a positive trend, and the effect estimate was in favour of the use of Baicalein, despite the variation in the concentration of Baicalein.

At the baseline analysis, the cross linkers did not show any favour/improvement in μTBS when compared to control.

Meta-analysis: (Figure 3 and Figure 4).

All the studies that performed ageing of the samples for more than 6 months were included under meta-analysis. The seven studies which satisfied the above criteria were Castellan et al., 2013 [1], Gerhardt et al., 2016 [20], Hass et al., 2016 [35], Monteiro et al., 2013 [21], Neri et al., 2016 [23], Venigalla et al., 2016 [19] and Zheng et al., 2015 [38]. Two meta-analyses with sub group analyses were performed on the μTBS after ageing. The first analysis considered seven studies with 14 datasets (four cross linkers), with 6–9 months of ageing (Figure 3) and the second analysis included two studies with seven datasets (two cross linkers) with a 12 months or more period of ageing (Figure 4). The studies were tested for subgroup differences (sub totals) and the pooled effect was not considered due to the various cross linkers used.

After 6 months of ageing (Figure 3), the overall pooled estimate for PA/GSE is appreciable and found to be evidenced of having a beneficial impact when compared to the control group (MD 8.29, 95% CI 3.13 to 13.45). The heterogeneity among the studies was high (I^2^ = 96%), signifying the great variation of effect size among the studies. The CSE, GTE and EGCG groups did not show an appreciable improvement in the bond strength which is evident from the pooled estimates. Bond strengths in the PA/GSE group, at 12 months of ageing (Figure 4), were significantly higher than in the control group (*p* < 0.00001) with moderate heterogeneity (I^2^ = 46%) among studies.

## 4. Discussion

The preservation of the collagen matrix integrity is indispensable for the long-term clinical success of composite restoration or to improve dentin bonding durability. Therefore, lower biodegradation rates and high mechanical properties of collagen are desirable. Considering the crucial role of MMPs in interfacial ageing over time, inhibiting the activity of host-derived MMPs and reducing the susceptibility of collagen matrices to MMP-induced degradation via cross-linking may thus be a rational and practical approach for the improvement of bonding durability [7].

Many techniques have been proposed for the complete infiltration of the resin monomers around the collagen fibres but it has always remained a challenge for researchers.

The use of low pH materials, such as acid etching in itself, is an ambiguous procedure, as it not only provides micromechanical retention but also activates the dormant dentinal MMPs. The cleavage of collagen occurs at the Gly–Leu/Isoleu peptide bond, between residues 775 and 776 [44,45]. The key point in preventing the degradation of collagen is to inhibit the binding of the enzyme at this specific site on the peptide chain. Two interventions have been proposed to achieve this stability. Direct deactivation of MMPs using MMP inhibitors is one of the efficient biochemical methods, such as the use of chlorhexidine which is a non-specific MMP inhibitor. Another efficient biochemical method is to crosslink the collagen and prevent it from degradation (biomodification), for example, glutaraldehyde and proanthocyanidin. When MMPs bind to collagen, there is unwinding of collagen thus allowing sufficient space to attack the specific Gly–Leu/Isoleu peptide bond. These cross linking agents prevent the unwinding of collagen and cross-linking of either the hemopexin-like or fibronectin-like domains also contribute to inactivation of the associated MMPs and reduction in their collagenolytic efficacy [7]. Cross linking does not improve the collagen’s tensile strength. However, when challenged by bacterial collagenase solution, the ultimate tensile strength of cross-linked collagen does not change, unlike the non-crosslinked collagen, thus proving beyond doubt that collagen crosslinking stabilises mechanical strength [46] thus improving the resistance to degradation by collagenases [47].

The synthetic MMP inhibitors proved to be not only cytotoxic but also lacked specificity [48]. The natural MMP inhibitors contain natural phytochemicals such as polyphenols (flavonoids) which stabilize and preserve the integrity of the collagen matrix (cross-linkers), thus reducing the biodegradation of collagen [28]. Thus there always has been an attempt to search for new molecular entities from natural resources [49].

The present systematic review analyzed the data from in vitro studies to assess different natural cross-linkers, on immediate and aged bond strength values (of at least 6 months and more). The studies that incorporated a crosslinker/MMP inhibitor within the adhesive composition [16,50]—ageing not specified or performed with thermocycling [17,24] and aged for less than six months [1,16,22,36]—were not included in the meta-analysis. It was observed that, regardless of the use of a natural cross-linker, there was considerable preservation of bond strength both immediately and after ageing, when compared to control. Thus the hypothesis was rejected. The overall result of this analysis showed that proanthocyanidin/Grape seed extract exhibits better bond strength values, especially after ageing.

The adhesion to tooth structure should ideally provide retentive strength, marginal seal, and clinical durability [51]. Hence, micro tensile bond strength is an ideal durability challenge to test the longevity of composite restorations and is also considered by the researchers as the best surrogate measure of dentin bond retention [52]. Another advantage of this method is that it permits the measurement of high bond strengths without cohesive failure of dentin [53]. Though many natural cross linkers have been studied, many did not adapt µTBS to study the durability, thus limiting the articles for systematic review.

This review witnessed a wide variation in the adhesives used for testing natural cross linkers—total etch (ethanol and acetone-based) and self etch. There was no remarkable observation made in the quantitative analysis to substantiate the superiority of one adhesive over the other. Ethanol wet bonding has been proposed to be one of the methods to prevent hydrolysis and improve bond durability [14,19], but ethanol used as a solvated primer did not improve its superiority over acetone in this analysis. Yet, in a study by Castellan [1], it was reported that acetone-based adhesive resulted in low bond strength values. Theoretically, cross-linker acts best on exposed collagen; therefore, sequential application of phosphoric acid, cross-linker, etch and rinse adhesive would be more effective. Zheng et al. [22] was the only study that tested the effect of total-etch and self-etch adhesives with GTE and reported no variation.

Distilled water and artificial saliva were the two commonly used ageing methods, and consensus could not be reached on the best ageing media due to the heterogeneity of the other parameters. According to Carrilho et al. [54], bond strengths were either preserved or increased in specimens when stored in oil, thus water-based media are ideal for storage/ageing.

A disparity in the duration of soaking of the sample/application of the test material, (cross-linker) ranged from 30 secs to 1 h. It was observed that 1 min of application showed favourable results and thus would be a constructive clinical step saving the chairside time. One hour of applying the MMPI/crosslinker [36,37] does not contribute any merit to a clinical situation and thus can be avoided in further studies.

The use of PA/GSE has no significant effect on immediate bond strength but after ageing for 6 and 12 months, the results of the meta analysis demonstrated significantly better bond strength compared to the control group. GSE is a rich PA source, which interacts and crosslinks with proteins with one of the four different mechanisms: Covalent interaction, ionic interaction, hydrogen bonding interaction and hydrophobic interaction [55]. Of the PA, 6.5% is the concentration tested by most of the researchers. Studies were also attempted to test the efficacy of PA mixed in adhesives on bond strength [16,22,39]. In the study by Epasinghe [16,39] 3% adhesive adversely affected the bond strength and in another study by Zheng [22], 5% PA in adhesive did not show any difference in bond strength after 24 h and 3 months. PA was also attempted in self etch adhesive [50] and had a similar bond strength to the control after ageing. PA 6.5% at 24 h did not show any significant increase in μTBS except for one study by Al Ammar [36], in which the bond strength of the GSE group was 71.06 and the control was 33.8. It can be assumed that the application of an active agent for 24 h could be the cause of such exaggerated results after 24 h. Largely after ageing, it was evident that the application of GSE/PA resulted in the preservation of bond strength.

CSE is also another source of PA, containing only 45% PA for the same amount of extract, whereas GSE includes 95%. The lower concentration of PA could probably be the reason for not producing evident results, i.e., there was no appreciable improvement in the bond strength in the studies included. Therefore, it cannot be a promising cross-linker.

Green tea (GTE), commonly known as Camellia sinesis, is a natural cross linker. This potential activity is mainly due to the polyphenols and catechins present [56]. Three studies evaluated the effect of green tea on μTBS and its effect after ageing. There was a wide variation in the concentrations studied, i.e., 2% [20], 1.1% [21] and 0.05% [22]. EGCG is a tea polyphenol. This catechin is claimed to stabilize the collagen chain and increase the number of collagen fibril cross-links [57]. Both the cross linkers (GTE and EGCG) did not show any appreciable increase in the bond strength after the ageing period, as was evident in the meta-analysis. This result could be due to the wide variation in the concentration of the cross linkers studied.

Baicalein, a flavonoid is derived from the roots of Scutellaria baicalensis. Its molecular structure is similar to PA, suggesting its similar inhibitory effects on endogenous MMP. However, the studies did not observe the effect of this cross linker after ageing and thus its long term effect could be analysed in the meta analysis.

The random effect model was adopted for meta analysis, taking into consideration the high heterogeneity (>50%) among the studies. The samples ranged from 5 to 41, consequently leading to high standard deviations and increased covariables. Thus, it would be more meaningful if the in vitro studies followed the same laboratory conditions and criteria to test the efficacy of cross linkers. Except for the different concentrations of the test materials, the following other factors can be standardized, such as: (a) having a control group (with similar treatment except for the test material); (b) a priori sample size calculation; (c) duration of the application of the test material; (d) selection of bonding agent and its application (total-etch and self-etch); (e) ageing period; (f) ageing media; and (g) blinding of the operator testing the samples. Doing so would not only reduce the bias but would lead to meaningful conclusions more scientifically. Unlike in the present review, it would avoid heterogeneity of the studies, making it simpler to compare one agent to another.

In this systematic review, only articles in English were selected and analyzed. The other limitation would be the criteria for choosing the in vitro studies. A low number of studies was included in the analysis as only a few studies met the inclusion criteria. The literature is abundant on natural crosslinker use, but only a few have tested μTBS after ageing. However, it is important to emphasize that in vitro bond strength tests are just one factor that can directly influence the efficacy of the dentin bonding and can be associated with clinical success or durability.

## 5. Conclusions

There was considerable heterogeneity across different protocols used, thus bringing some limitations in the meta-analysis approach. The available evidence indicates that GSE/PA, beyond doubt, is the most efficient natural cross-linker to date, in preserving the bond strength even after ageing. The other natural cross-linkers/MMPIs did not show such an improvement in the bond strength compared to the control after ageing, but it is evident that they may not impair the bond strength. A few studies individually did show some improvement, but when compared by way of the meta-analysis, it was observed as a pooled effect that other crosslinkers did not show any appreciable improvement in bond strength except for GSE/PA.

Further in vitro studies with long term ageing periods and standard protocols can be conducted to thoroughly understand the influence of the natural cross-linkers on resin bond durability. These studies should be followed by long term clinical trials to understand their effect on the clinical success of the restorations.

## Figures and Tables

**Table 1:** The 5 cross linkers and their concentrations assessed in this systematic review.

Sl No.	Cross Linker	Concentration	References
**1.**	GSE/PA	6.5% 5% 1% 2% 3% in adhesive	[1,17,19,35,36,37] [38] [39]
**2.**	CSE	6.5%	[1,37]
**3.**	GTE	2% 1.1% 0.05%	[20] [22] [21]
**4.**	EGCG	2% 0.1%	[20] [23]
**5.**	Baicalein	2.5 μg/mL 3.125 μmol/L, 12.5 μmol/L, 6.25 μmol/L	[11] [24]

(GSE/PA—Grape seed extract/Proanthocyanidin; CSE—Cocoa seed extract; GTE—Green tea extract; EGCG—Epigallocatechin Gallate).

**Table 2:** The characteristics of in vitro studies included.

Author	Year	Natural MMP Inhibitors Studied	Adhesive Used	Other Test Materials Tested	Soaking Period (in the Test Material)	Tested Interval (Aging)	Primary Outcome	Secondary Outcome	Included in Meta Analysis
Al Ammar [36]	2009	6.5% Grape seed extract in PBS	Acetone and Ethanol based (Total etch)	0.5% Genipin 5% Glutaraldehyde	1 h	24 h (distilled water)	Micro tensile bond strength (μ TBS)	Fracture pattern	No
Castellan [37]	2010	6.5% Grape seed extract (distilled water) 6.5% Cocoa seed extract	Acetone and Ethanol based (Total etch)		60 min 10 min	24 h (distilled water)	μ TBS	Modulus of elasticity swelling ratio	No
Epasinghe [39]	2012	Proanthcynidine in adhesive at 1%,2% and 3%	Ethanol based (Total etch)		30 s	24 h (distilled water)	μ TBS	Failure modes and Nano leakage	No
Castellan [1]	2013	6.5% Grape Seed extract (distilled water) 6.5% Cocoa Seed extract (ethanol–Acetone solvents)	Acetone and Ethanol based (Total etch)		10 min	24 h 3 months 6 months 12 months (Artificial saliva)	μ TBS		Yes
Monteiro [21]	2013	Green tea extract 1.1%	Ethanol based (total etch)	CHX	60 s	24 h 6 months	μ TBS	Failure modes	Yes
Zheng [22]	2015	Green tea extract 0.05%	Ethanol based (Total etch) & Self Etch	CHX FeSO_4_ Galardin	60 s	9 months Artificial Saliva	μ TBS	failure modes	Yes
Gajjela [17]	2016	6.5% Grape seed extract (distilled water)	Self etch	Riboflavin/Chitosan CHX	10 min	Not mentioned	μ TBS		No
Hass [35]	2016	6.5% PA	Ethanol based (Total etch)	UVA Riboflavin Glutaraldehyde	60 s	24 h 18 months Artificial saliva	μ TBS	failure modes nano leakage DC with in Hybrid layer In situ zymography Cytotoxicity evaluation	Yes
Neri JR [23]	2016	0.1% EGCG	Self etch	CHX 2%	60 s	24 h 6 months 12 months (distilled water)	μ TBS	failure modes	Yes
Gerhardt [20]	2016	Green tea extract 2% EGCG 2%	Self etch	CHX 2%	60 s	24 h 6 months (distilled water)	μ TBS	failure modes	Yes
Venigella [19]	2016	6.5% PA	Ethanol based (Total etch)	Riboflavin carbodiimide	2 min	24 h 6 months (distilled water)	μ TBS	failure modes	Yes
Zheng [38]	2017	5% PA	Total etch	Chlorhexidine Doxycycline	30 s	24 h 3 months	μ TBS	Immunolabeling of MMPs Micro permeability assessment	No
J Li [11]	2018	2.5 μg/mL Baicalein	Ethanol based (Total etch)	5% GD 1% DMSO	2 min	Immediate 3 months 6 months	μ TBS	Degree of conversion, Gelatinolytic and collagenolytic activity evaluation, Failure mode analysis, Interfacial nano leakage testing	No
Poyi chu [24]	2019	Baicalein (3.125 μmol/L, 12.5 μmol/L, 6.25 μmol/L)	Ethanol based (Total etch)		2 min	Immediately Ageing with thermocycling	μ TBS	Cell viability assay, Cell cycle analysis, Gene expression analysis, western blot for protein expression analysis	No

**Table 3:** Risk of bias of studies considering characteristics reported in the material and methods section.

Sl No.	Study	Teeth Randomization	Teeth Free of Caries	Specimens with Similar Dimensions	Materials Used According to Manufacture Instructions	Sample Size Calculation	Blinding of the Operator of the Testing Machine	Sample Preparation and Handling	Application of Test Material	Specimen Test According to Standard Specifications	Risk of Bias
**1.**	CASTELLAN 2013 [1]	N	Y	Y	Y	N	N	Y	y	Y	medium (6)
**2.**	HASS 2016 [35]	Y	Y	Y	N	N	N	Y	Y	Y	medium (6)
**3.**	VENIGELLA 2016 [19]	Y	Y	Y	N	N	N	Y	y	Y	medium (6)
**4.**	GERHARDT 2016 [20]	Y	Y	Y	Y	N	N	Y	Y	Y	Low (7)
**5.**	MONTEIRO 2013 [21]	Y	Y	Y	Y	N	N	Y	Y	Y	Low (7)
**6.**	ZHENG 2014 [38]	Y	Y	Y	Y	N	N	Y	Y	Y	Low (7)
**7.**	NERI JR 2016 [23]	Y	Y	Y	Y	N	N	Y	Y	Y	Low (7)

Articles that reported one to four items—High risk of bias, Five to six items—Medium risk of bias, Seven to nine items—Low risk of bias.

## Data Availability

The data will be shared upon a reasonable request to the corresponding author.
